# JIB-04, a Pan-Inhibitor of Histone Demethylases, Targets Histone-Lysine-Demethylase-Dependent AKT Pathway, Leading to Cell Cycle Arrest and Inhibition of Cancer Stem-Like Cell Properties in Hepatocellular Carcinoma Cells

**DOI:** 10.3390/ijms23147657

**Published:** 2022-07-11

**Authors:** Jina Lee, Ji-Soo Kim, Hye-In Cho, So-Ra Jo, Yeun-Kyu Jang

**Affiliations:** 1 Department of Systems Biology, College of Life Science and Biotechnology, Yonsei University, Seoul 120-749, Korea; apurebluesky@gmail.com (J.L.); j_7475@naver.com (J.-S.K.); hyeincho@hanmail.net (H.-I.C.); whthfkw@gmail.com (S.-R.J.); 2 BK21 Yonsei Education & Research Center for Biosystems, Yonsei University, Seoul 120-749, Korea

**Keywords:** histone lysine demethylases (KDMs), hepatocellular carcinoma, cancer stem cells, AKT2, AKT2/FOXO3a/p21/RB axis, cell cycle arrest, histone lysine demethylase inhibitor, JIB-04

## Abstract

JIB-04, a pan-histone lysine demethylase (KDM) inhibitor, targets drug-resistant cells, along with colorectal cancer stem cells (CSCs), which are crucial for cancer recurrence and metastasis. Despite the advances in CSC biology, the effect of JIB-04 on liver CSCs (LCSCs) and the malignancy of hepatocellular carcinoma (HCC) has not been elucidated yet. Here, we showed that JIB-04 targeted KDMs, leading to the growth inhibition and cell cycle arrest of HCC, and abolished the viability of LCSCs. JIB-04 significantly attenuated CSC tumorsphere formation, growth, relapse, migration, and invasion in vitro. Among KDMs, the deficiency of *KDM4B*, *KDM4D*, and *KDM6B* reduced the viability of the tumorspheres, suggesting their roles in the function of LCSCs. RNA sequencing revealed that JIB-04 affected various cancer-related pathways, especially the PI3K/AKT pathway, which is crucial for HCC malignancy and the maintenance of LCSCs. Our results revealed KDM6B-dependent *AKT2* expression and the downregulation of E2F-regulated genes via JIB-04-induced inhibition of the AKT2/FOXO3a/p21/RB axis. A ChIP assay demonstrated JIB-04-induced reduction in H3K27me3 at the *AKT2* promoter and the enrichment of KDM6B within this promoter. Overall, our results strongly suggest that the inhibitory effect of JIB-04 on HCC malignancy and the maintenance of LCSCs is mediated via targeting the KDM6B-AKT2 pathway, indicating the therapeutic potential of JIB-04.

## 1. Introduction

Liver cancer is one of the leading causes of cancer-related deaths globally [1]. Among all primary liver cancers, hepatocellular carcinoma (HCC) is the most common subtype, accounting for approximately 78% of the total cases [2]. The prognosis of patients with HCC largely depends on the tumor stage. Early-stage HCC can be treated using surgical resection, transplantation, and ablation techniques using radiofrequency [1]. However, in many cases, HCC is diagnosed at an advanced stage, thereby leading to a very poor survival rate [3,4]. A better understanding of the mechanisms underlying HCC malignancy and metastasis may contribute to the development of more efficacious therapeutic strategies primarily aimed at treating advanced-stage HCC.

Recently, many studies have reported that cancer stem cells (CSCs) are responsible for tumor growth, metastasis, and recurrence [5]. Furthermore, CSCs have been identified in various cancer types, including melanoma, colon, liver, pancreas, breast, and ovarian cancers [6,7,8,9,10,11]. More importantly, CSCs are considered to be the major cause of recurrence, as they are highly resistant to conventional therapies, such as chemotherapy and radiation [12,13,14]. Therefore, the eradication of liver cancer stem cells (LCSCs) is expected to ultimately improve the survival rate in patients with liver cancer.

JIB-04 is a pan-inhibitor that targets the demethylase activity of the Jumonji family of histone lysine demethylases (KDMs). This small molecule was also found to be a potent anti-cancer agent against several cancers, such as lung and prostate cancer cells [15], and Ewing sarcoma [16]. Interestingly, JIB-04 inhibited the growth of drug-resistant cells, including temozolomide-resistant glioblastoma [17] and taxane-platin-resistant lung cancer cells [18]. A recent study from our laboratory reported that colorectal CSCs could be selectively targeted by JIB-04 [19]. Thus, these results suggest that JIB-04 may be helpful in curing aggressive and malignant cancers. Although recent studies have pointed out the inhibitory effect of JIB-04 on the growth of drug-resistant brain cancer and lung cancer cells, as well as the self-renewal of colorectal CSCs, there is currently no experimental evidence to support that JIB-04 selectively targets liver CSCs and inhibits the malignancy of hepatocellular carcinoma cells.

Many lines of evidence support the concept that AKT plays a key role in cancer cell development and tumorigenesis [20,21]. AKT2 is highly expressed in many human cancers, including non-small cell lung cancer [22], colorectal cancer [23], and thyroid cancer [24]. In colon cancer, *AKT2* overexpression affected radiation sensitivity and the DNA repair system [25], while AKT2 played a role in chemotherapy sensitivity and cancer cell survival in non-small cell lung cancer [26]. Intriguingly, the relationship between the PI3K/AKT pathway and CSCs was recently revealed in esophageal cancer [27]. In addition, PTEN/PI3K/AKT signaling was reported to be important for the maintenance of prostate cancer stem-like cells [28]. This experimental evidence supports the functional link between the PI3K/AKT signaling pathway and cancer stem cell function.

In the present study, we investigate the effect of JIB-04 on the maintenance of liver CSCs and the malignancy of HCC cells. Our data demonstrate that JIB-04 treatment results in growth inhibition and cell cycle arrest in liver cancer cells and reduces the viability of tumorsphere cells derived from LCSCs. The reduced expression of CSC marker genes and the inhibition of the growth and relapse of tumorspheres are observed in JIB-04-treated HCC cells. More importantly, our results show that a deficiency of *KDM4B*, *KDM4D*, and *KDM6B* reduces the viability of tumorspheres. Intriguingly, our data reveal that the JIB-04-dependent suppression of E2F target genes contributes to cell cycle arrest via negative control of the KDM6B-AKT2-FOXO-RB regulatory circuit. Furthermore, *AKT2* gene expression is reduced by both JIB-04 treatment and KDM6B depletion. Collectively, our results suggest that JIB-04-mediated targeting of histone demethylases, such as KDM4B, KDM4D, and KDM6B, inhibits the malignancy of hepatocellular carcinoma via growth inhibition and cell cycle arrest and, in addition, affects the maintenance and viability of liver CSCs.

## 2. Results

### 2.1. JIB-04 Caused Reduced Cell Proliferation and Cell Cycle Arrest in HCC Cells

**Figure 1:**
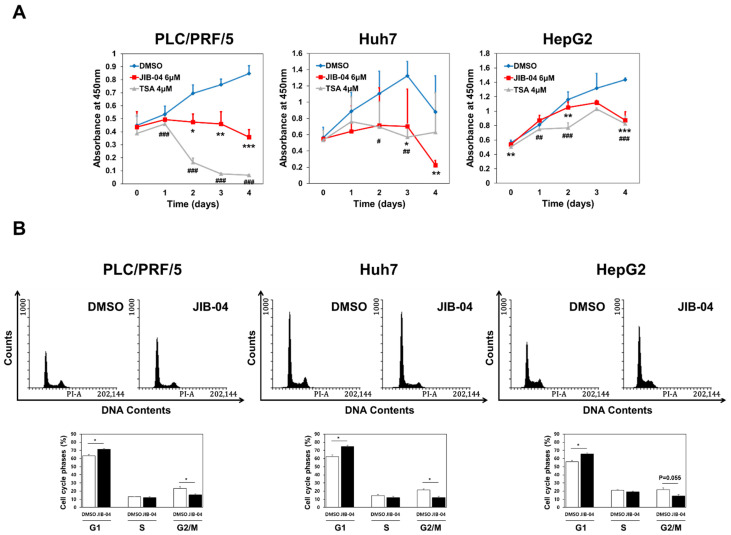
JIB-04 inhibits cell proliferation in a time-dependent manner and causes G_1_-phase cell-cycle arrest. (**A**) The viability of HCC cells cultured after treatment with DMSO (mock control), 6 μM JIB-04, or 4 μM TSA for indicated intervals was analyzed by CCK assay. The error bars indicate standard deviation; *n* = 3 in PLC/PRF/5, Huh7, and HepG2 cells. (**B**) (*Top panels*) Representative histograms of the cell cycle phase distribution in PLC/PRF/5, Huh7, and HepG2 cells after treatment with DMSO (control) or 6 μM JIB-04 for 24 h. Cells were stained with propidium iodide to detect their DNA content. (*Bottom panels*) Bar graphs representing relative cell populations in cell cycle phases G_1_, S, and G_2_/M. The data demonstrate JIB-04-dependent effects on cell cycle phases. Data are represented as mean ± SEM derived from triplicate measurements. * *p* < 0.05, ** *p* < 0.01, and *** *p* < 0.001 (DMSO vs. JIB-04); # *p* < 0.05, ## *p* < 0.01, and ### *p* < 0.001 (DMSO vs. TSA positive control).

Because our previous report showed the effect of JIB-04 on the viability and cell cycle of human colorectal cancer cells [19], we attempted to determine if JIB-04 could influence those of human HCC cells. When three different human HCC lines, namely PLC/PRF/5, Huh7, and HepG2, were treated with 6 μM JIB-04 for 4 days, the viability of all three cell lines was significantly decreased in a time-dependent manner compared with DMSO-treated controls (Figure 1A). The effect of JIB-04 on cell viability was comparable to that of trichostatin A (TSA), which was used as a control drug because of its known ability to target liver CSCs [29,30]. In addition, we investigated the effect of JIB-04 on the cell cycle progression in HCC cells using a FACS analysis to assess whether the reduced cell viability (Figure 1A) was related to any defects in the cell cycle. Compared with DMSO-treated controls, all the JIB-04-treated HCC cells showed increased G_1_-phase subpopulations and decreased G_2_/M-phase subpopulations (Figure 1B), suggesting the occurrence of G_1_/S arrest in JIB-04-treated HCC cells. Thus, these data suggested that the reduced cell viability in JIB-04-treated HCC cells might be partly caused by JIB-04-induced cell cycle defects.

### 2.2. JIB-04 Treatment Interrupted HCC Cell Migration and Invasion

**Figure 2:**
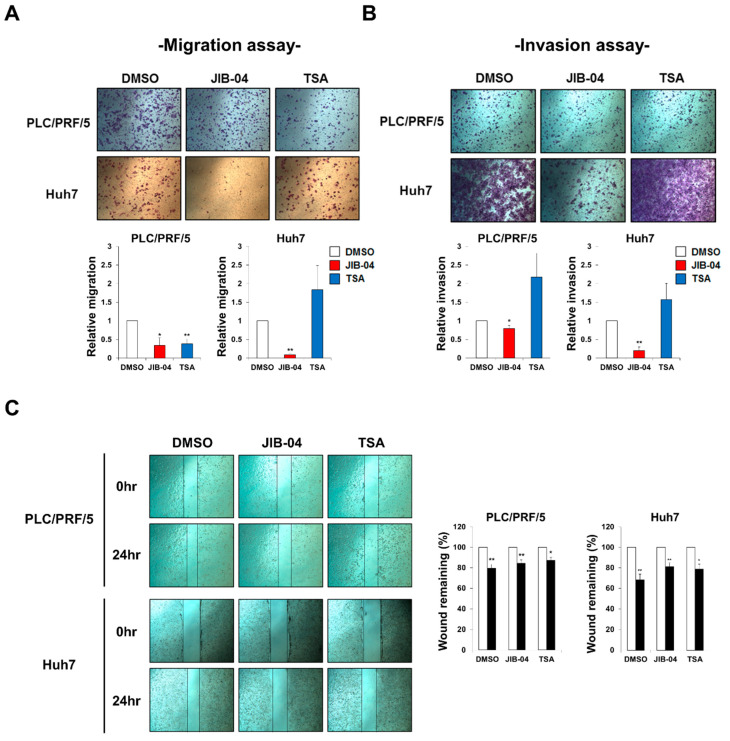
JIB-04 reduces the capacity of cell migration and invasion in HCC cells. (**A**) Cell migration was investigated with transwell assays after treatment with DMSO, 6 µM JIB-04, or 4 µM TSA for 24 h (*n* = 3). (**B**) Invasive ability was examined in transwells coated with Matrigel after treatment with DMSO, 6 µM JIB-04, or 4 µM TSA for 24 h (PLC/PRF/5 (*n* = 4); Huh7 (*n* = 3)). (**C**) Cell migration was assessed with a wound-healing assay. Cells were scraped with a yellow pipette tip and treated with DMSO, 6 µM JIB-04, or 4 µM TSA for 24 h. Cells were imaged under a light microscope after injury. Cell migration was quantified by measuring remaining wound areas before and after treatment with DMSO, 6 µM JIB-04, or 4 µM TSA. Data are represented as mean ± SEM of triplicate measurements. * *p* < 0.05, ** *p* <0.01 compared with DMSO-treated controls.

We carried out transwell assays to determine whether JIB-04 affected the migratory and invasive abilities of HCC cells. PLC/PRF/5 and Huh7 cells treated with JIB-04 displayed reduced migration and invasion compared with control cells treated with DMSO or TSA (Figure 2A,B). Moreover, the effect of JIB-04 on cell migration was further confirmed using a wound-healing assay. Consistent with the data depicted in Figure 2A, the migratory ability of both PLC/PRF/5 and Huh7 cells was decreased upon JIB-04 treatment compared with that of the DMSO-treated control cells (Figure 2C). Therefore, these results demonstrated that JIB-04 inhibits the migratory and invasive capacities of HCC cells in vitro.

### 2.3. JIB-04 Treatment Reduced the Expression Levels of CSC Markers

**Figure 3:**
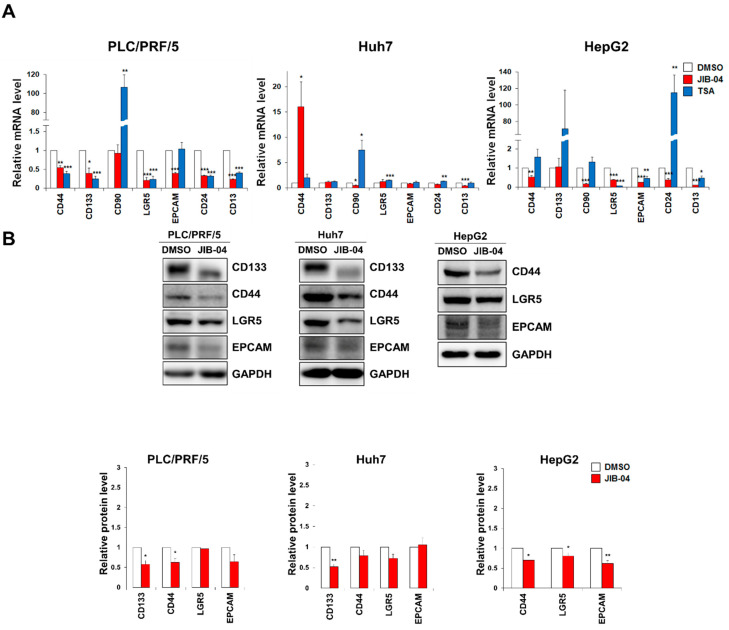
The expression levels of CSC markers in HCC cells are decreased by JIB-04. (**A**) The mRNA expression of cancer stem cell marker genes was analyzed using qRT-PCR in PLC/PRF/5, Huh7, and HepG2 cultures after treatment with DMSO (control), 6 μM JIB-04, or 4 μM TSA (positive control) for 24 h. All data are normalized to GAPDH and plotted relative to the expression level in control cells. Data are represented as mean ± SEM derived from triplicate measurements (*n* = 3); * *p* < 0.05, ** *p* < 0.01, and *** *p* < 0.001. (**B**) (***Top** panels*) Protein levels of CD133, CD44, LGR5, and EpCAM in HCC cells after treatment with 6 μM JIB-04 for 48 h were confirmed with Western blot analysis. GAPDH was used as a loading control. (***Bottom** panels*) Quantification based on densitometry of Western blotting data from top panels in (**B**). All data are normalized to GAPDH. Data are represented as mean ± SEM of triplicate measurements; * *p* < 0.05 and ** *p* < 0.01.

To assess the effect of JIB-04 on the stemness of liver CSCs, we investigated the mRNA expression levels of liver CSC marker genes, including *CD44*, *CD133*, *CD90*, *LGR5*, *EpCAM*, *CD24*, and *CD13*, in three HCC cell lines, namely PLC/PRF/5, Huh7, and HepG2, after JIB-04 treatment. Our qRT-PCR data showed that the mRNA expression levels of CSC marker genes were generally reduced in all three JIB-04-treated cell lines compared to DMSO-treated control cells, implying a significant reduction in the liver CSC population following JIB-04 treatment (Figure 3A). Next, we analyzed the protein levels of CSC markers in JIB-04-treated HCC cells. A Western blot analysis demonstrated that the protein levels of CD44, LGR5, and EpCAM were decreased in all three HCC cell lines.

CD133 protein levels were downregulated in PLC/PRF/5 and Huh7 cells (Figure 3B). In addition, fluorescent immunocytochemistry showed that the expression levels of CSC markers were decreased in PLC/PRF/5 and Huh7 cells after JIB-04 treatment (Supplementary Figure S1). Together, our data indicate that JIB-04 treatment affected the expression of CSC marker genes, suggesting its potential role in the anti-LCSC activity.

### 2.4. JIB-04 Diminished the Tumor Initiation, Growth, and Relapse Abilities of Tumorspheres Derived from Liver CSCs

**Figure 4:**
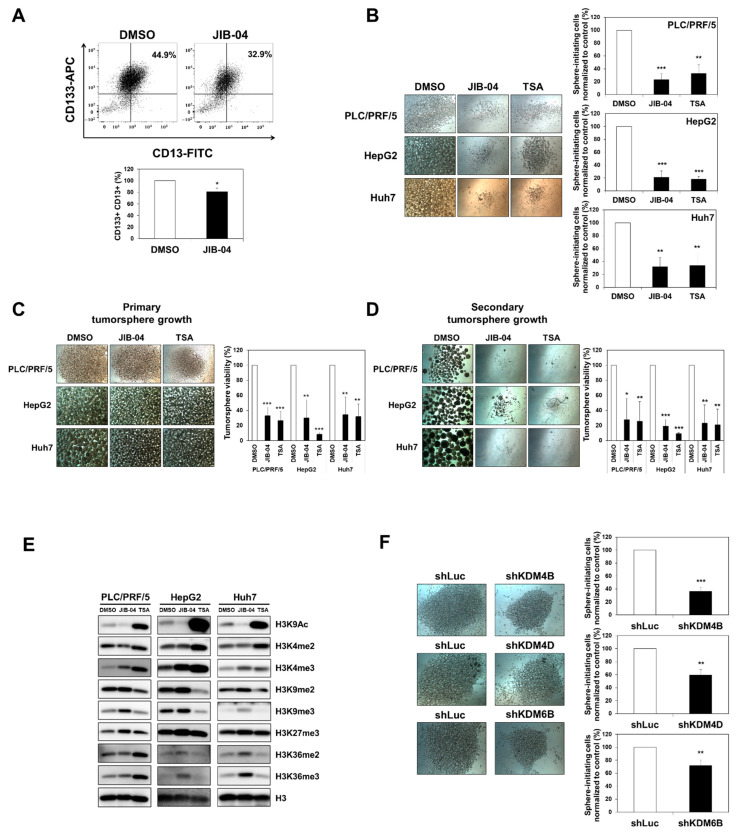
JIB-04 impairs the maintenance and viability of cancer stem-like cells in HCC cells. (**A**) CD133^+^/CD13^+^ subpopulations in PLC/PRF/5 cells after treatment with DMSO or 6 µM JIB-04 for 24 h were analyzed with flow cytometry analysis. Treatment with JIB-04 resulted in a decreased percentage of CD133^+^/CD13^+^ subpopulations compared with DMSO (control). Data are mean ± SEM of triplicate measurements; * *p* <0.05, ** *p* < 0.01, and *** *p* < 0.001. (**B**) Phase contrast image of tumorspheres derived from cells cultured with DMSO (control), 6µM JIB-04, or 4µM TSA for 24 h (***Left** panel*). Percentage of sphere-initiating cells measured using CCK assay (***Right** panel*). The number of DMSO-treated cells was set as 100 (n = 3). The data indicated that JIB-04 reduces tumor-initiating ability in HCC cells. (**C**) Phase contrast image of primary tumorspheres after treatment with DMSO (control), 6µM JIB-04, or 4µM TSA for 2 days (***Left** panel*). Viability of primary tumorspheres measured using CCK assay (***Right** panel*). The number of DMSO-treated cells was set as 100 (n = 3). (**D**) Phase contrast image of secondary tumorspheres developed from primary tumorspheres without drug treatment for 12 days. Viability of secondary tumorspheres measured with CCK assay (***Right** panel*). The number of DMSO-treated cells was set as 100 (n = 3). (**E**) Western blot analysis of histone methylation levels after treatment with DMSO or 6µM JIB-04 for 24 h. H3 was used as a loading control. (**F**) The tumor-initiating ability of CSCs in PLC/PRF/5 cells was decreased due to the deficiency in KDM4B, KDM4D, and KDM6B. Phase contrast image of tumorspheres derived from KDM4B-, KDM4D-, or KDM6B-depleted knockdown cells (***Left** panel*). Percentage of sphere-initiating cells measured with CCK assay (***Right** panel*). The number of DMSO-treated cells was set as 100 (*n* = 3). Data are represented as mean ± SEM of triplicate measurements; * *p* < 0.05, ** *p* < 0.01, and *** *p* < 0.001.

Many lines of evidence support that CSCs enable themselves to initiate and drive tumorigenesis, which may contribute to the chance of relapse. First, we focused on the CD133^+^/CD13^+^ population in HCC cells, as CD133^+^/CD13^+^ hepatocytes are known to possess CSC characteristics [29]. We investigated the effect of JIB-04 treatment on CD133^+^/CD13^+^ cells using a FACS analysis. Our FACS data showed that the CD133^+^/CD13^+^ population was lower in JIB-04-treated cells than that in DMSO-treated control cells (Figure 4A), suggesting the potency of JIB-04 as a selective drug for targeting liver CSCs. Furthermore, we performed tumorsphere formation assays to investigate the effect of JIB-04 on tumor initiation, growth, and relapse. Since CSCs can form tumorspheres in serum-free culture medium on non-adherent culture dishes, tumorsphere formation assays are very useful for studying the stem-cell-like properties of CSCs. To confirm whether JIB-04 affected stem-cell-like properties such as tumor initiation, growth, and relapse abilities, we established an LCSC sphere-forming culture system under three-dimensional culture conditions, as previously described [19]. To begin, the effect of JIB-04 on the tumor initiation ability of CSCs was evaluated using three different HCC cell lines. We cultured PLC/PRF/5, Huh7, and HepG2 tumorspheres on non-adherent culture dishes after treatment with DMSO, 6 μM JIB-04, or 4 μM TSA for 24 h. After 7 days, the DMSO-treated control cells developed dense and round-shaped tumorspheres; however, the JIB-04-treated cells failed to form tumorspheres, remaining almost as tiny spheres (Figure 4B, left panel). Quantitative analysis with a cell-counting kit (CCK) assay demonstrated that JIB-04 treatment resulted in a decrease in the percentage of sphere-initiating cells to about 20% of that of control cells and was comparable to the TSA treatment in inhibiting the initiation of tumorspheres (Figure 4B, right panel). Furthermore, to evaluate the ability of JIB-04 to diminish tumorsphere growth, we induced tumorsphere formation for 5 days and subsequently treated the spheres with DMSO (mock control), 6 μM JIB-04, or 4 μM TSA for 2 days. The size of JIB-04-treated tumorspheres was found to have reduced (Figure 4C, left panel). Consistent with these morphological changes, CCK assays further supported that JIB-04 treatment reduced the viability of tumorsphere cells, suggesting that JIB-04 inhibited the growth of the tumorspheres by targeting CSCs (Figure 4C, right panel). To assess the effect of JIB-04 on relapse, we developed secondary tumorspheres by culturing the surviving cells shown in Figure 4C (left panel) in a standard stem cell medium without additional drug treatment for 12 days. The control cells derived from mock-treated primary tumorspheres were able to form secondary tumorspheres comparable to the primary tumorspheres. In contrast, the surviving cells derived from JIB-04-treated primary tumorspheres mostly failed to develop secondary tumorspheres (Figure 4D, left panel). As evident from the CCK assays, the number of viable cells in the secondary tumorspheres derived from JIB-04-treated primary tumorspheres was also reduced compared with that observed for DMSO-treated controls, implying that the regrowth of tumorspheres was efficiently suppressed by pretreatment with JIB-04 (Figure 4D, right panel).

As mentioned earlier, JIB-04 is a pan-inhibitor of Jumonji histone demethylases [15]. The global levels of several histone modifications in colorectal cancer cells were found to be influenced by JIB-04 treatment [19]. To confirm the effect of JIB-04 on global histone H3 modification levels in human HCC cells, we performed a Western blot analysis. As expected, we found that JIB-04 upregulated the tri-methylation of H3K4, as well as the di- and tri-methylation of H3K36, which are known as active chromatin markers (Figure 4E). In addition, the di- and tri-methylation of H3K9, as well as the tri-methylation of H3K27, which are hallmarks of inactive chromatin, were increased in JIB-04-treated HCC cells (Figure 4E). Thus, these data suggested that JIB-04 inhibits the KDM4 and KDM6 histone demethylase families in HCC cells.

Based on the data above, we attempted to identify the histone demethylase directly related to the JIB-04-based inhibition of tumorsphere formation. To accomplish this, we investigated whether a deficiency in histone demethylases could influence the tumorsphere formation ability of liver CSCs. Tumorsphere formation assays were performed using various histone-demethylase-depleted knockdown cells. Our results demonstrated that the sphere-forming abilities of *KDM4B*-, *KDM4D*-, and *KDM6B*-knockdown cells were significantly decreased compared to that of the *shLuc* control knockdown cells (Figure 4F). In contrast, the CCK assay showed that the viabilities of tumorsphere cells in *KDM4A*-, *KDM4C*-, and *KDM6A*-depleted cells were increased compared to that in the control knockdown cells (Supplementary Figure S2). Taken together, our data suggested that JIB-04 exerts anti-LCSC effects via the inhibition of KDM4B, KDM4D, and KDM6B.

### 2.5. Transcriptome Analysis Revealed JIB-04-Targeted Pathways in HCC Cells

To uncover the mechanism by which JIB-04 affects the malignancy of HCC cells and liver CSCs, we performed an RNA-sequencing analysis using RNA extracts from PLC/PRF/5 cells after treatment with 6 μM JIB-04 for 24 h. JIB-04 treatment resulted in the upregulation of 700 genes and the downregulation of 459 genes (Supplementary Figure S3A). A Kyoto Encyclopedia of Genes and Genomes (KEGG) pathway analysis revealed that JIB-04 treatment resulted in an altered expression of genes involved in the cell cycle, apoptosis, and cellular senescence that were also related to several cancers, including HCC (Supplementary Figure S3B). JIB-04 also altered the expression of genes involved in various signaling pathways, such as the FOXO signaling pathway, the MAPK signaling pathway, the Wnt signaling pathway, and the PI3K-Akt signaling pathway (Supplementary Figure S3B). Among these genes, we focused on genes related to the cell cycle and the PI3K-Akt signaling pathway because the PI3K-Akt pathway is known to contribute to cancer progression via the regulation of G_1_/S cell cycle transition, as well as the maintenance and viability of cancer stem-like cells [27,28,30,31].

### 2.6. JIB-04 Induced Anti-Cancer Effects by Targeting AKT-FOXO3a-p21-RB-E2F Axis

**Figure 5:**
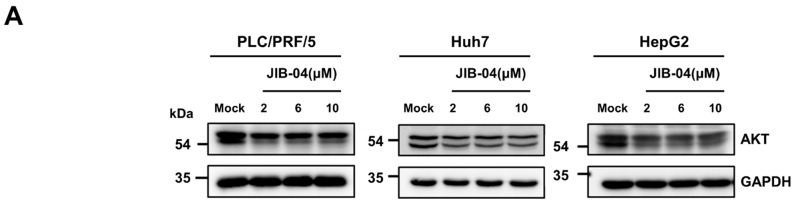

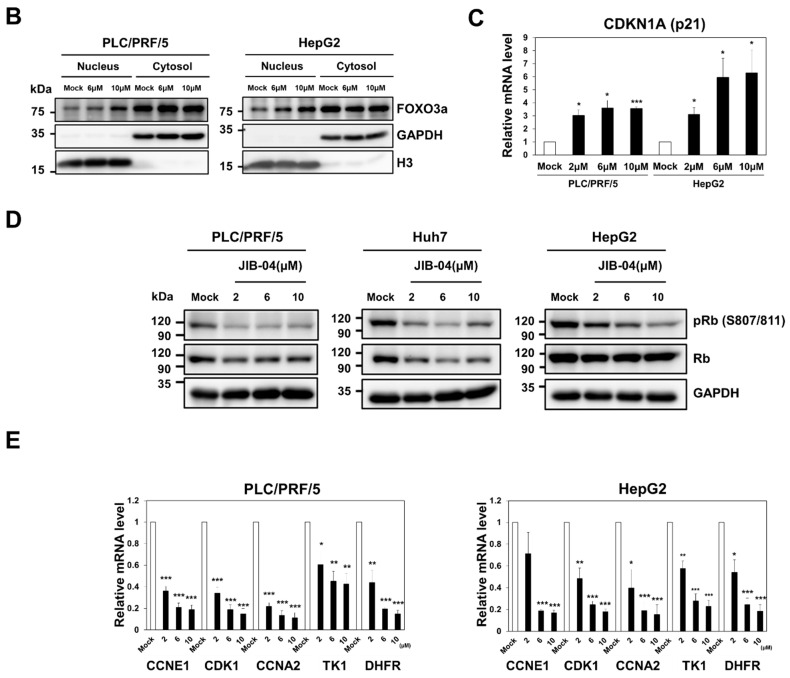
JIB-04 blocks cell cycle progression by regulating the AKT/FOXO/RB axis. (**A**) Protein expressions of AKT in three different HCC cell lines after treatment with increasing doses of JIB-04 or DMSO (control) for 24h. GAPDH was used as a loading control. (**B**) Nucleocytoplasmic shuttling of FOXO3a in PLC/PRF/5 and HepG2 cells after treatment with increasing doses of JIB-04 or DMSO (control) for 24h. Nucleus and cytosolic fractions were separately isolated and subjected to Western blot analysis using an anti-FOXO3a antibody. GAPDH was used as a cytosolic marker, and H3 was used as a nuclear marker. (**C**) The mRNA expression levels of *CDKN1A* (*p21*) in PLC/PRF/5 and HepG2 cells after treatment with increasing doses of JIB-04 or DMSO (control) were analyzed using qRT-PCR. All data are normalized to GAPDH and plotted relative to the expression level in control cells. Data are represented as mean ± SEM of triplicate measurements; * *p* < 0.05, and *** *p* < 0.001. (**D**) The levels of the phosphorylated form and total RB protein in three different HCC cell lines after treatment with increasing doses of JIB-04 or DMSO (control) for 24h were examined with Western blot analysis. GAPDH was used as a loading control. (**E**) The mRNA expression levels of cell-cycle-related genes, such as *CCNE1, CDK1, CCNA2, TK1*, and *DHFR*, in PLC/PRF/5 and HepG2 cells after treatment with increasing doses of JIB-04 or DMSO (control) for 24h were analyzed using qRT-PCR. All data are normalized to GAPDH and plotted relative to the expression level in control cells. Data are represented as mean ± SEM of triplicate measurements; * *p* < 0.05, ** *p* < 0.01, and *** *p* < 0.001.

Based on our findings, we next questioned whether the PI3K-Akt signaling pathway was responsible for JIB-04-induced G_1_/S cell cycle arrest in HCC cell lines, as shown in Figure 1B. To achieve this, AKT protein levels in HCC cell lines after treatment with DMSO or JIB-04 were first analyzed with a Western blotting analysis using a pan-total AKT antibody. As shown in Figure 5A, JIB-04 treatment reduced AKT protein levels in a dose-dependent manner. We then investigated the nuclear translocation of FOXO proteins in JIB-04-treated HCC cells because the AKT-dependent phosphorylation of FOXO factors results in their cytoplasmic accumulation and subsequent degradation, thereby eliminating their transactivation capacity [32,33]. To determine whether JIB-04 caused the nuclear translocation of FOXO3a, we performed a cell fractionation analysis in PLC/PRF/5 and HepG2 cell lines after treatment with DMSO or JIB-04. In JIB-04-treated PLC/PRF/5 and HepG2 cells, the treatment induced the accumulation of FOXO3a in the nucleus in a dose-dependent manner (Figure 5B). Since FOXOs control cell cycle progression through the transcriptional regulation of cyclin-dependent kinase (CDK) inhibitors such as p21, p27, and p15 [34,35,36,37], we assumed the increased expression in CDK inhibitors by the JIB-04-induced nuclear enrichment of FOXOs. Our qRT-PCR analysis revealed that the mRNA expression of the *p21*(*CDKN1A*) gene was induced by JIB-04 treatment, but the expressions of p15 and p27 did not increase (Figure 5C, Supplementary Figure S4). Finally, we investigated RB activation and the expression of E2F target genes because p21 can lead to the activation of RB and subsequently cause the suppression of E2F-mediated transcription in the nucleus [38,39]. A Western blot analysis revealed that JIB-04 treatment reduced the phosphorylation levels of RB protein, indicating RB activation (Figure 5D). In addition, the qRT-PCR assay showed that the mRNA expressions of E2F target genes in JIB-04-treated PLC/PRF/5 and HepG2 cell lines were significantly decreased in a dose-dependent manner, suggesting RB-mediated inactivation of the E2F transcription factor (Figure 5E). Thus, these results suggested that the AKT-FOXO3a-p21-RB-E2F axis is involved in JIB-04-mediated anti-cancer effects.

### 2.7. KDM6B Regulated AKT2 Expression through H3K27me3 Modifications

**Figure 6:**
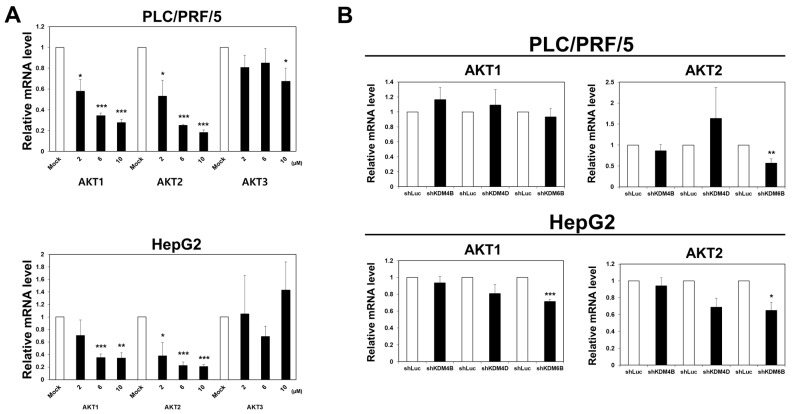

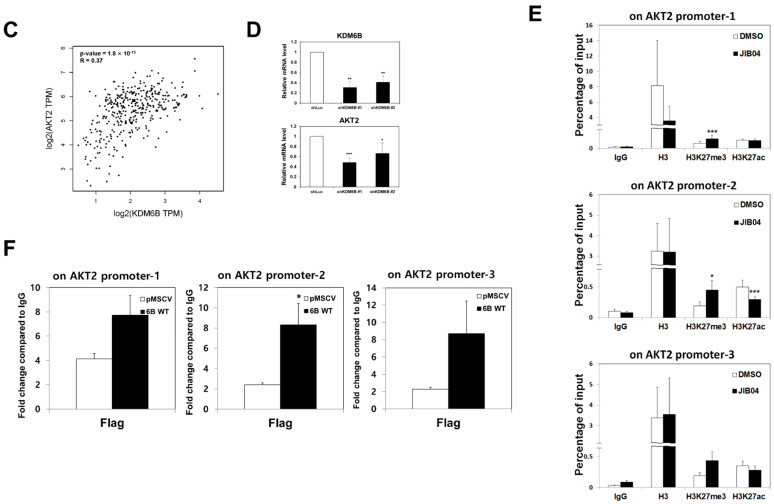
KDM6B is required for transcriptional activation of the *AKT2* gene in HCC cells. (**A**) The mRNA expression levels of *AKT1*, *AKT2*, and *AKT3* in PLC/PRF/5 and HepG2 cells after treatment with increasing doses of JIB-04 or DMSO (control) for 24h were analyzed using qRT-PCR (AKT1 and AKT2 (*n* = 3); AKT3 (*n* = 4)). (**B**) The mRNA expressions of *AKT1* and *AKT2* in *KDM4B*-, *KDM4D*-*,* and *KDM6B*-depleted HCC cells (PLC/PRF/5: *AKT1* (*n* = 4) and *AKT2* (*KDM4B*-depleted cells *n* = 5; *KDM4D*- and *KDM6B*-depleted cells *n* = 4); HepG2: *AKT1* (*KDM4B*-depleted cells *n* = 5, *KDM4D*-depleted cells *n* = 6, and *KDM6B*-depleted cells *n* = 3) and *AKT2* (*KDM4B*-depleted cells *n* = 5, *KDM4D*-depleted cells *n* = 6, and *KDM6B*-depleted cells *n* = 3). (**C**) Pair-wise correlation between *KDM6B* and *AKT2* using the Spearman method. The plot was obtained from the GEPIA database. (**D**) The mRNA expression levels of *KDM6B* and *AKT2* in *KDM6B*-depleted PLC/PRF/5 cells were analyzed using qRT-PCR (*n* = 4). (**E**) The enrichment of epigenetic markers in *AKT2* promoter regions of HepG2 cells after treatment with DMSO (control) or 6 µM JIB-04 for 24 was analyzed with ChIP assay (*n* = 4). (**F**) The occupancy of *KDM6B* protein in the promoter regions of the *AKT2* gene was examined by ChIP assays. Data are represented as mean ± SEM of triplicate measurements; * *p* < 0.05, ** *p* < 0.01, and *** *p* < 0.001.

Since JIB-04 treatment reduced the expression of AKT protein (Figure 5A), we hypothesized that the JIB-04-induced inhibition of certain histone demethylases may cause the downregulation of the *AKT* gene. Therefore, we investigated the expressions of AKT family genes (*AKT1, AKT2*, and *AKT3*) in JIB-04-treated PLC/PRF/5 and HepG2 cells. The qRT-PCR analysis revealed that the mRNA expressions of *AKT1* and *AKT2* were decreased upon JIB-04 treatment, but it was not altered for the *AKT3* gene (Figure 6A). To determine which histone demethylase was related to this AKT transcriptional regulation, we investigated the mRNA expression levels of *AKT1* and *AKT2* in *KDM4B*-, *KDM4D*-, and *KDM6B*-knockdown cells. Our data showed that only *KDM6B* depletion significantly decreased the *AKT2* mRNA expression in both the PLC/PRF/5 and HepG2 cell lines (Figure 6B). Moreover, correlation analysis indicated that the expressions of *AKT2* and *KDM6B* were positively correlated (Figure 6C). In addition, decreased *AKT2* mRNA levels were observed in two different *KDM6B*-depleted cell lines (Figure 6D). These results suggested that KDM6B is responsible for *AKT2* gene regulation.

Since KDM6B is known to be responsible for erasing the tri-methylation of lysine-27 on histone H3 (H3K27me3) [40], we investigated whether JIB-04 treatment influenced the H3K27me3 modification level at the *AKT2* gene promoter in PLC/PRF/5 cells. Our ChIP assay revealed that the H3K27me3 levels at the *AKT2* gene promoter were increased by JIB-04 treatment, whereas the H3K27ac levels were reduced (Figure 6E). These data suggested that the increased levels of H3K27me3 by JIB-04 treatment may contribute to the downregulation of *AKT2* expression. To determine whether KDM6B could associate directly with the *AKT* gene promoter, we performed a ChIP assay using PLC/PRF/5 cells stably expressing an empty vector or FLAG-tagged KDM6B. The ChIP assay confirmed that KDM6B protein was significantly enriched in the promoter region of *AKT2* (Figure 6F). Collectively, these results suggested that KDM6B is involved in the transcriptional activation of *AKT2* via the downregulation of H3K27me3 at the *AKT2* gene promoter.

## 3. Discussion

Recently, the small molecule JIB-04, a pan-inhibitor of Jumonji demethylases, has been shown to inhibit growth and induce apoptosis in drug-resistant glioblastoma and lung cancer cells [17,18]. In addition, we previously reported that JIB-04 targeted colorectal cancer stem cells (CSCs) via the selective inhibition of the Wnt/β-catenin signaling pathway [19]. Although HCC is the most common subtype of liver cancer with a poor prognosis and its high recurrence rate is correlated with the presence of liver CSCs [41,42], the role of JIB-04 in the malignancy of HCC and liver CSC function has not been elucidated. In this study, we identified a novel role of JIB-04 in the viability and maintenance of liver CSCs, as well as in the cell cycle progression of HCC cells, using three different HCC cell lines.

As described previously [19], JIB-04 largely induced G_2_/M cell cycle arrest in colorectal cancer cells and preferentially eradicated colorectal CSCs by inhibiting the Wnt/β-catenin signaling pathway. In contrast, JIB-04 caused G_1_/S cell cycle arrest in HCC cells, and the KDM–AKT2 pathway was found to be important for cell cycle progression in HCC cells. In this study, the effect of JIB-04 on the tumorigenicity of liver CSCs was assessed using a tumorsphere formation assay, as described elsewhere [43,44]. Similar to the role of JIB-04 in colorectal CSC function, JIB-04 treatment resulted in the downregulation of several CSC marker genes, as well as the inhibition of the tumor-initiating ability of liver CSCs. Interestingly, the growth and relapse of secondary tumorspheres derived from the primary tumorspheres after pre-treatment with JIB-04 were significantly abolished, as shown in Figure 4D. More importantly, tumorsphere formation was reduced by deficiencies in KDM4B, KDM4D, and KDM6B. Thus, these results suggested that the JIB-04-mediated effect on liver CSC function was mediated by targeting histone demethylases such as KDM4B, KDM4D, and KDM6B. Moreover, our data suggested that the JIB-04-dependent inhibition of KDMs directly or indirectly affected the expression of CSC marker genes, including *CD133*, *CD44*, *CD90*, *LGR5*, *EpCAM*, *CD24*, and *CD13*. Taken together, our results indicated that JIB-04 is a promising candidate for improving the survival rate in patients with liver cancer by targeting liver CSCs, as the CSC markers are known to be responsible for chemoresistance, metastasis, and tumor relapse [10,45,46,47,48,49,50,51].

In this study, we found that the tri-methylation levels of H3K4, H3K36, H3K9, and H3K27 were globally increased in JIB-04-treated HCC cells (Figure 4E). The patterns of JIB-04-induced histone H3 methylation in HCC cells were similar to those observed in JIB-04-treated colorectal cancer cells, but the increase in H3K27me3 levels in JIB-04-treated HCC cells was more prominent than that observed in JIB-04-treated colorectal cancer cells. To understand the effect of JIB-04 on the malignancy of HCC and the function of LCSCs, we aimed to determine the KDM(s) responsible for the JIB-04-treatment-related phenotypes in HCC cells. As previously reported in an interesting study [15], JIB-04 can inhibit the demethylase activity of JMJD2A (KDM4A), JMJD2B (KDM4B), JMJC2C (KDM4C), JMJD2D (KDM4D), JMJD2E (KDM4E), JARID1A (KDM5A), and JMJD3 (KDM6B), implying the potential role of the KDM4, KDM5, and KDM6 families in JIB-04-related phenotypes. Based on these results, we propose that KDM4B, KDM4D, and KDM6B are involved in the cell growth and cell cycle progression of HCC cells, as well as in the survival and maintenance of LCSCs. In particular, our data highlighted the importance of KDM6B in HCC malignancy and functions of LCSCs because the tumor-initiating ability of LCSCs was reduced by KDM6B deficiency (Figure 4F), and the JIB-04-induced elevation of H3K27me3 levels could be reduced by the demethylase activity of KDM6B [15].

Although most of the data in this study supported the concept that KDM6B may be one of the main targets underlying the effect of JIB-04 on HCC malignancy and LCSC function, we needed to understand the mechanism of action of KDM6B in the JIB-04-related phenotypes of HCC cells. Therefore, RNA-sequencing analysis was performed to uncover various cancer-related signaling pathways, including the FOXO signaling pathway, the MAPK signaling pathway, the HIF-1 signaling pathway, and the PI3K-AKT signaling pathway (Supplementary Figure S3). Among these, we focused on the PI3K-AKT signaling pathway because it is known to be involved in both the regulation of cell cycle progression [52] and the development of many cancer types, including HCC [53]. Interestingly, it has been reported that the PI3K-AKT pathway plays an important role in CSC biology [54,55]. Consistent with the clues derived from the genome-wide analyzed data, our results showed that JIB-04 significantly decreased AKT protein levels and subsequently affected the FOXO3a-p21-RB-E2F axis, the downstream regulatory cascade of AKT (Figure 5).

AKT is an evolutionarily conserved serine-protein kinase that regulates numerous signaling pathways by controlling downstream effectors that are required for the maintenance of cell homeostasis [56]. Three AKT isoforms, namely AKT1, AKT2, and AKT3, play a crucial role in cellular functions such as transcription, protein synthesis, cell cycle progression, and cell death regulation [21,56,57,58,59]. Interestingly, aberrant AKT expression was observed in various human cancers [60], and the relationship between AKT and cancer stem cells has also been revealed [26,27,61,62]. Of note, among the three AKT isoforms, the mRNA expression levels of *AKT1* and *AKT2* were downregulated by JIB-04 treatment, as shown in Figure 6A. *AKT2* expression was significantly affected only by KDM6B deficiency, revealing AKT2 as a potential target of KDM6B (Figure 6B,D). Given this, it is important to understand how KDM6B regulates *AKT2* expression. Since KDM6B showed demethylase activity specific for the tri-methylation of histone H3K27, it was speculated that KDM6B positively regulates *AKT2* gene transcription via the removal of inactive chromatin markers, such as H3K27me3. As expected, our ChIP assay revealed a significant increase in the H3K27me3 levels at *AKT2* promoter regions and the further enrichment of KDM6B within the chromatic domains of the *AKT2* promoter, as shown in Figure 6E,F. Thus, we concluded that KDM6B could associate directly with the chromatin domains at *AKT2* promoter regions and remove the tri-methylation of histone H3K27, an inactive chromatin marker, from the *AKT2* promoter, leading to increased *AKT2* expression. Subsequently, the KDM6B-mediated upregulation of *AKT2* was required for cell cycle progression via the regulatory cascade of the AKT2-FOXO3a-p21-RB-E2F axis, as was evident from the experimental analyses carried out in this study. Moreover, the KDM6B-AKT2 pathway is known to be involved in the survival and maintenance of liver CSCs, as previously has been described in several studies [26,61,62].

In summary, in this study, we presented a plausible model to explain how JIB-04 treatment induced growth inhibition and cell cycle arrest in HCC cells, as well as the impairment of liver CSC functions. JIB-04 interfered with the cell cycle progression of HCC cells and cancer stem-like cell properties via the AKT2-FOXO3a-RB axis by inhibiting the histone demethylase activity of KDM6B on the *AKT2* promoter. Overall, our results suggested that strategies targeting the KDM6B-AKT2 pathway with JIB-04, a pan-histone demethylase inhibitor, may contribute to the development of therapeutics against liver cancer.

## 4. Materials and Methods

### 4.1. Cell Culture and Drug Treatment

PLC/PRF/5, HepG2, and Huh7 cells were maintained in a humidified incubator at 37 °C in an atmosphere of 5% CO_2_. PLC/PRF/5 cells were cultured in RPMI-1640 medium (Welgene, Gyeongsan, Korea) supplemented with 10% (*v*/*v*) fetal bovine serum (FBS; Atlas Biologicals, Fort Collins, CO, USA) and 1× penicillin-streptomycin solution (Cat. #30–002-cl; Corning, Mediatech Inc., Manassas, VA, USA). HepG2 and Huh7 cells were cultured in DMEM (Welgene) containing 10% (*v*/*v*) FBS (Atlas Biologicals) and 1× penicillin-streptomycin solution (Cat. #30–002-cl; Corning, Mediatech Inc.). For drug treatment, JIB-04 (Cat. #15338; Cayman Chemical, Ann Arbor, MI, USA) and trichostatin A (TSA; Cayman Chemical, Ann Arbor, MI, USA) were individually dissolved in dimethyl sulfoxide (DMSO) and diluted before use. The PLC/PRF/5 cell line was provided kindly by Dr. Injae Shin (Yonsei University, Seoul, Korea), and the HepG2 and Huh7 cell lines by Dr. Wang Shick Ryu (Yonsei University, Korea). All cell lines were tested for mycoplasma contamination.

### 4.2. Lentiviral Production and Infection

KDM4B-, KDM4D-, and KDM6B-depleted HCC cells were generated using shRNA-based gene silencing using lentiviral production and infection. First, shRNAs were cloned into a pLKO.1 TRC cloning vector (Addgene, Watertown, MA, USA). The shRNAs targeted the coding sequences and 3′-untranslated regions. shRNAs targeting firefly luciferase genes were used as a control. Then, to generate lentivirus, 293FT was transfected with 3 µg pLKO.1 shRNA vector, 2.25 µg pMD2.G and pMD2.G envelope plasmid, and 6.75 μg psPAX2 using lipofectamine 2000 (Thermo Fisher, Waltham, MA, USA). The viral supernatant was collected and used to infect HCC cells. Finally, to knockdown KDM4B, KDM4D, and KDM6B, HCC cells were infected with pLKO.1 viral particles with 6 µg/mL polybrene overnight. After 24 h, the infected cells were selected with 2.5 µg/mL puromycin [63].

### 4.3. Retroviral Production and Infection

For retrovirus production, either pMSCV empty vector or pMSCV-based FLAG-KDM6B was used to transfect 293FT with 4.5 μg of gag-pol and 0.5 μg of VSVG. The viral supernatant was collected and used to infect HCC cells 48 h after transfection. PLC/PRF/5 cells were infected with retrovirus overnight with 6 μg/mL polybrene. After 24 h, the infected cells were selected with 2.5 µg/mL puromycin [64].

### 4.4. Cell Growth Assay

Cell proliferation was measured using a cell-counting kit (CCK-8 assay kit; Dojindo Corporation, Kumamoto, Japan). Twenty-four hours prior to experiments, cells were plated in the wells of a 96-well plate and treated with either DMSO, JIB-04, or TSA for 24, 48, 72, or 96 h. CCK-8 solution was then added to 100 μL of culture medium, and the cells were incubated at 37 °C for 2 h. Optical density was measured at 450 nm using a microplate reader (Sunrise-Basic Tecan, Tecan Austria GmbH, Grodig, Austria).

### 4.5. Cell Cycle Analysis

Approximately 8 × 10^5^ cells were seeded in a 60-pie dish. After 24 h, the cells were treated with 6 μM JIB-04, and the dish was incubated for 24 h. The cells were then fixed, stained with propidium iodide, and analyzed using flow cytometry (FACS), as described previously [19].

### 4.6. Western Blotting 

A Western blot analysis was performed as described previously [65]. Briefly, cells were treated with either vehicle (DMSO), JIB-04, or TSA for 48 h and then were harvested by centrifugation. The pellets were resuspended in radioimmunoprecipitation assay (RIPA) buffer comprising 150 mM NaCl, 50 mM Tris (pH 8.0), 1% NP-40, 0.5% sodium deoxycholate, 0.1% sodium dodecyl sulfate (SDS), and protease inhibitors and were incubated on ice. Supernatants containing proteins of interest were collected after centrifugation. The protein concentration was determined using a Bradford assay. For Western blot analysis, proteins were separated with 10% SDS-PAGE and transferred to polyvinylidene fluoride (PVDF) membranes (EMD Millipore, Billerica, MA, USA). The membranes were treated with primary antibodies, including CD133 (Cat. #130-092-395; Miltenyi Biotec, Bergisch Gladbach, Germany), CD44 (Cat. #sc9960; Santa Cruz Biotechnology, Dallas, TX, USA), leucine-rich repeat-containing G-protein-coupled receptor 5 (LGR5, Cat. #NBP1-28904; Novus Biologicals, Centennial, CO, USA), epithelial cell adhesion molecule (EPCAM, Cat. #2929; Cell Signaling Technology, Danvers, MA, USA), Akt (pan) (Cat. #4691S; Cell Signaling), Rb (Cat. #9309; Cell Signaling), Phospho-Rb (Ser807/811) (Cat. #9308; Cell Signaling), FoxO3a (Cat. #2497; Cell Signaling), and glyceraldehyde-3-phosphate dehydrogenase (GAPDH, Cat. # AbC-2003; AbClon Inc., Guro-gu, Korea).

### 4.7. Fluorescent Immunocytochemistry

For the fluorescent immunocytochemistry assay, HCC cells were cultured on coated coverslips in 6-well plates. After 24 h, HCC cells were treated with JIB-04 for 48 h. The cells were fixed with 3.7% paraformaldehyde and treated with 0.2% Triton X-100 for permeabilization. Then, the cells were incubated with CD133, CD44, and GAPDH primary antibodies. After 24 h, the cells were further incubated with secondary antibodies (Alexa goat anti-mouse) with DAPI for 2 h. Confocal image acquisition was performed using a Carl Zeiss confocal microscope.

### 4.8. Chromatin Isolation Assay

Chromatin isolation was performed to separate the nuclear and cytoplasmic fractions of the cells. The cells were harvested, and the pellets were resuspended in hypotonic buffer containing 1% NP40, 5 mM MgCl_2_, 10 mM NaCl, 20 mM Tris (pH8.0), and protease inhibitors and were incubated on ice for 1 h. The lysate was centrifuged at 14,000 rpm (4 °C) for 25 min, and the supernatant was collected as the cytosolic fraction. The pellet was lysed with hypertonic buffer containing 1% NP40, 5 mM MgCl_2_, 150 mM NaCl, 20 mM Tris (pH8.0) and was sonicated to efficiently isolate the nuclei. The sonicated pellet was centrifuged at 14,000 rpm (4 °C) for 25 min, and the supernatant was collected as the nuclear fraction.

### 4.9. ChIP Assay

A ChIP assay was performed using a SimpleChIP Enzymatic chromatin IP Kit ChIP kit (Cell Signaling #9002S) according to the manufacturer’s instructions. Briefly, cells were cross-linked in situ by the addition of 37% formaldehyde to a final concentration of 1%, incubated at room temperature for 10 min, and then were incubated with glycine for 5 min. Chromatin was digested and immunoprecipitated using IgG (Cell Signaling #2729), H3 (Cell Signaling #4620), H3K27me3 (Cat. #ab6002; Abcam), and H3K27ac (Cat. #ab4729; Abcam) antibodies overnight at 4 °C. The purified DNA was used for PCR amplification using primers specific to promoter fragments of the AKT2 gene [66].

### 4.10. Isolation of RNA and Quantitative Reverse Transcription-Polymerase Chain Reaction (qRT-PCR) Analysis

qRT-PCR analysis was performed as described previously [63]. Total RNA was isolated using TRI-Reagent (Cat. #TR118; Molecular Research Center, Inc., Cincinnati, OH, USA). Expression levels were analyzed by qRT-PCR with SYBR Premix Ex Taq II (Takara Bio, Shiga, Japan) and a 7300 Real-Time PCR system (Applied Biosystems, Franklin Lakes, NJ, USA) using primer sets for target genes. qRT-PCR was performed with the following primers: human CD133, (sense) 5′-TGCAGTGGATCGAGTTCT-3′ and (antisense) 5′-TCCTATGCCAAACCAAAA-3′; human CD44, (sense) 5′-GGGGGTCCCATACCACTC-3′ and (antisense) 5′-CCAAGAGGGATGCCAAGA-3′; human CD90, (sense) 5′-CCCGTGAGACAAAGAAGCA-3′ and (antisense) 5′-TGCTGGTGAAGTTGGTTCG-3′; human LGR5, (sense) 5′-CCTGTCCTTGCCTGTGCT-3′ and (antisense) 5′-CCACCCTGAGCAACATCC-3′; human EpCAM, (sense) 5′- AGCTGGCCGTAAACTGCTT-3′ and (antisense) 5′- GCCAGCTTTGAGCAAATGA-3′; human CD24, (sense) 5′-CCCACGCAGATTTATTCCAG-3′ and (antisense) 5′- ACCACGAAGAGACTGGCTGT-3′; human CD13, (sense) 5′-CCCGATTCCAATGTTACCC-3′ and (antisense) 5′- TCATTGCCTGATGTGCTGA-3′; and human GAPDH, (sense) 5′-TGATGACATCAAGAAGGTGGTGAAG-3′ and (antisense) 5′- TCCTTGGAGGCCATGTGGGCCAT-3′.

### 4.11. Cell Migration and Invasion Assay

Transwell cell migration assays were performed using Falcon cell number inserts (Cat. #353097; Corning, Corning, NY, USA). Cells were pre-incubated with the indicated drugs for 24 h, after which 7 × 10^4^ cells were placed in the insert in serum-free medium and allowed to migrate for 2 days. The outer chamber was filled with 750 µL of medium containing 10% FBS. After incubation, non-migrating cells on the upper surface of the insert were removed using a cotton swab. Migrating cells were fixed and stained with crystal violet (Sigma-Aldrich, St. Louis, MO, USA). For the invasion assay, 2 × 10^5^ cells were seeded in Matrigel-coated inserts and allowed to invade for 2 days. Invasive cells were stained as described above.

### 4.12. Wound-Healing Assay

HCC cells were cultured in 12-well plates at 3.6 × 10^5^ cells to form confluent monolayers. Wounds were created using a sterile pipette tip after the HCC cells were cultured for 24 h. The wounded areas were recorded at 0 h and 24 h and were measured using ImageJ software.

### 4.13. Tumorsphere Formation

Tumorspheres derived from cancer cells have been proved to display characteristics of CSCs. CSCs are significant causes of metastasis and drug resistance [67]. Tumorspheres are grown in serum-free and non-adherent conditions. Only cancer stem cells can survive and proliferate in these conditions. Therefore, the formation of tumorspheres is a helpful method for the enrichment of CSCs [68].

To perform the tumor initiation ability of liver CSCs, we treated PLC/PRF/5, HepG2, and Huh7 cells with DMSO, 6 µM JIB-04, or 4 µM TSA for 24 h or 48h and then seeded 1 × 10^4^ of the treated cells in stem cell medium (DMEM: F12, 20 ng/mL EGF, 20 ng/mL FGF, 5 µg/mL insulin, and 1× B27 supplement) on ultra-low-attachment six-well plates (Corning). After 7 days, we detected tumorspheres using a cell-counting kit (CCK)-8 from Dojindo according to the manufacturer’s protocol.

To examine the effect of JIB-04 on tumor growth, we cultured primary tumorspheres for 5 days and then treated them with DMSO, 6 µM JIB-04, or 4 µM TSA for 2 days. We then measured the viability of the tumorspheres using a CCK assay. To examine the effect of JIB-04 on tumor recurrence, we trypsinized primary tumorspheres and re-seeded them without drug treatment to produce secondary tumorspheres. After 12 days, we measured the viability of the secondary tumorspheres using a CCK assay [19].

### 4.14. Histone Preparation 

Histones were prepared as described previously [19]. Briefly, histones were extracted from cell lysates using H_2_SO_4_ and precipitated in trichloroacetic acid. After washing the precipitated histones with acetone, they were dissolved in distilled water, and the protein content was determined using a Bradford assay. The following antibodies were used for Western blotting: H3K9Ac (Cat. #07-352; Millipore, Billerica, MA, USA), H3K4me2 (Cat. #07-030; Millipore), H3K4me3 (Cat. #ab8580; Abcam, Cambridge, UK), H3K9me2 (Cat. #ab1220; Abcam), H3K9me3 (Cat. #07-442; Millipore), H3K27me3 (Cat. #ab6002; Abcam), H3K36me2 (Cat. #07-274; Upstate), H3K36me3 (Cat. #ab9050; Abcam), and H3 (Cat. #ab1791; Abcam).

### 4.15. FACS Double-Staining

The expression profiles of CD13 and CD133 in PLC/PRF/5 cells were analyzed using flow cytometry. PLC/PRF/5 cells were harvested and washed with cold FACS buffer (1× PBS with 2% FBS and 0.09% sodium azide). The cells were incubated with FcR-blocking reagent (Miltenyi Biotec, 130-059-901) for 10 min in cold FACS buffer to block non-specific Fc interactions. CD13-FITC antibody (eBioscience, 11-0138-42) and CD133-APC antibody (Miltenyi Biotec, 130-113-184) were incubated with cells in the dark at 4 °C. After 1 h, labeled cells were washed with FACS buffer and analyzed using a BD FACS LSR II SORP system.

### 4.16. RNA Sequencing 

RNA sequencing was conducted using Macrogen Inc. (Seoul, Korea). RNA extracts from PLC/PRF/5 cells treated with DMSO or 6 µM JIB-04 for 24 h were subjected to cDNA library construction using a TruSeq Stranded mRNA LT Sample Prep Kit (Illumina, San Diego, CA, USA). The samples were checked for quality using FastQC v0.11.5 software and subjected to sequencing using a HiSeq 4000 sequencer (Illumina). The Kyoto Encyclopedia of Genes and Genomes (KEGG) database was used to determine the pathways of differentially expressed genes. The pathways were ranked using Fisher’s exact test with a threshold of significance set by *p*-value. The data discussed in this publication were deposited in NCBI’s Gene Expression Omnibus and are accessible under GEO series accession number GSE179345.

### 4.17. Gene Correlation Analysis

Gene Expression Profiling Interactive Analysis (GEPIA) is an online TCGA-based tool used to analyze RNA sequencing expression data. Pearson’s correlation analysis for KDM6B and AKT2 was performed using GEPIA.

### 4.18. Statistical Analysis

Data are expressed as means ± SEM or means ± SD. The data between controls and experimental groups were analyzed with two-tailed Student’s *t*-tests. Significance levels were set as follows: * *p* < 0.05, ** *p* < 0.01, and *** *p* < 0.001.

Raw data were used in Western blot figures.

## Data Availability

Not applicable.

## Supplementary Materials

The following supporting information can be downloaded at: https://www.mdpi.com/article/10.3390/ijms23147657/s1.

## References

[1] Forner A., Reig M., Bruix J. (2018). Hepatocellular carcinoma. Lancet.

[2] Laursen L. (2014). A preventable cancer. Nature.

[3] Bruix J., Sherman M. (2005). Management of hepatocellular carcinoma. Hepatology.

[4] Ye S., Takayama T., Geschwind J., Marrero J.A., Bronowicki J. (2010). Current Approaches to the Treatment of Early Hepatocellular Carcinoma. Oncologist.

[5] Visvader J.E., Lindeman G.J. (2012). Cancer stem cells: Current status and evolving complexities. Cell Stem Cell.

[6] Li C., Heidt D.G., Dalerba P., Burant C.F., Zhang L., Adsay V., Wicha M., Clarke M.F., Simeone D.M. (2007). Identification of pancreatic cancer stem cells. Cancer Res..

[7] O’Brien C.A., Pollett A., Gallinger S., Dick J.E. (2007). A human colon cancer cell capable of initiating tumour growth in immunodeficient mice. Nature.

[8] Phi L.T.H., Sari I.N., Yang Y.G., Lee S.H., Jun N., Kim K.S., Lee Y.K., Kwon H.Y. (2018). Cancer stem cells (CSCs) in drug resistance and their therapeutic implications in cancer treatment. Stem Cells Int..

[9] Schatton T., Murphy G.F., Frank N.Y., Yamaura K., Waaga-Gasser A.M., Gasser M., Zhan Q., Jordan S., Duncan L.M., Weishaupt C. (2008). Identification of cells initiating human melanomas. Nature.

[10] Yang Z.F., Ho D.W., Ng M.N., Lau C.K., Yu W.C., Ngai P., Chu P.W.K., Lam C.T., Poon R.T.P., Fan S.T. (2008). Significance of CD90+ Cancer Stem Cells in Human Liver Cancer. Cancer Cell.

[11] Zhang S., Balch C., Chan M.W., Lai H.C., Matei D., Schilder J.M., Yan P.S., Huang T.H.M., Nephew K.P. (2008). Identification and characterization of ovarian cancer-initiating cells from primary human tumors. Cancer Res..

[12] Sukowati C.H.C., Rosso N., Crocè L.S., Tiribelli C. (2010). Hepatic cancer stem cells and drug resistance: Relevance in targeted therapies for hepatocellular carcinoma. World J. Hepatol..

[13] Bao S., Wu Q., McLendon R.E., Hao Y., Shi Q., Hjelmeland A.B., Dewhirst M.W., Bigner D.D., Rich J.N. (2006). Glioma stem cells promote radioresistance by preferential activation of the DNA damage response. Nature.

[14] Li X., Lewis M.T., Huang J., Gutierrez C., Osborne C.K., Wu M.F., Hilsenbeck S.G., Pavlick A., Zhang X., Chamness G.C. (2008). Intrinsic resistance of tumorigenic breast cancer cells to chemotherapy. J. Natl. Cancer Inst..

[15] Wang L., Chang J., Varghese D., Dellinger M., Kumar S., Best A.M., Ruiz J., Bruick R., Peña-Llopis S., Xu J. (2013). A small molecule modulates Jumonji histone demethylase activity and selectively inhibits cancer growth. Nat. Commun..

[16] Parrish J.K., McCann T.S., Sechler M., Sobral L.M., Ren W., Jones K.L., Tan A.C., Jedlicka P. (2018). The Jumonji-domain histone demethylase inhibitor JIB-04 deregulates oncogenic programs and increases DNA damage in Ewing Sarcoma, resulting in impaired cell proliferation and survival, and reduced tumor growth. Oncotarget.

[17] Banelli B., Daga A., Forlani A., Allemanni G., Marubbi D., Pia Pistillo M., Profumo A., Romani M. (2017). Small molecules targeting histone demethylase genes (KDMs) inhibit growth of Temozolomide-resistant glioblastoma cells. Oncotarget.

[18] Dalvi M.P., Wang L., Zhong R., Kollipara R.K., Park H., Bayo J., Yenerall P., Zhou Y., Timmons B.C., Rodriguez-Canales J. (2017). Taxane-Platin-Resistant Lung Cancers Co-develop Hypersensitivity to JumonjiC Demethylase Inhibitors. Cell Rep..

[19] Kim M.S., Cho H.I., Yoon H.J., Ahn Y.-H., Park E.J., Jin Y.H., Jang Y.K. (2018). JIB-04, A Small Molecule Histone Demethylase Inhibitor, Selectively Targets Colorectal Cancer Stem Cells by Inhibiting the Wnt/βCatenin Signaling Pathway. Sci. Rep..

[20] Hers I., Vincent E.E., Tavaré J.M. (2011). Akt signalling in health and disease. Cell. Signal..

[21] Jiang N., Dai Q., Su X., Fu J., Feng X., Peng J. (2020). Role of PI3K/AKT pathway in cancer: The framework of malignant behavior. Mol. Biol. Rep..

[22] Liu T., Zhu J., Du W., Ning W., Zhang Y., Zeng Y., Liu Z., Huang J.A. (2020). AKT2 drives cancer progression and is negatively modulated by miR-124 in human lung adenocarcinoma. Respir. Res..

[23] Rychahou P.G., Kang J., Gulhati P., Doan H.Q., Chen L.A., Xiao S.Y., Chung D.H., Evers B.M. (2008). Akt2 overexpression plays a critical role in the establishment of colorectal cancer metastasis. Proc. Natl. Acad. Sci. USA.

[24] Ringel M.D., Hayre N., Saito J., Saunier B., Schuppert F., Burch H., Bernet V., Burman K.D., Kohn L.D., Saji M. (2001). Overexpression and overactivation of Akt in thyroid carcinoma. Cancer Res..

[25] Sahlberg S.H., Gustafsson A.S., Pendekanti P.N., Glimelius B., Stenerlöw B. (2014). The influence of AKT isoforms on radiation sensitivity and DNA repair in colon cancer cell lines. Tumor Biol..

[26] Lu Z., Wang M., Wu S., Ye M., Lin Z., Shun T., Duan C. (2018). Microrna-137-regulated akt serine/threonine kinase 2 inhibits tumor growth and sensitizes cisplatin in patients with non-small cell lung cancer. Oncol. Lett..

[27] Honjo S., Ajani J.A., Scott A.W., Chen Q., Skinner H.D., Stroehlein J., Johnson R.L., Song S. (2014). Metformin sensitizes chemotherapy by targeting cancer stem cells and the mTOR pathway in esophageal cancer. Int. J. Oncol..

[28] Dubrovska A., Kim S., Salamone R.J., Walker J.R., Maira S.-M., García-Echeverría C., Schultz P.G., Reddy V.A. (2009). The role of PTEN/Akt/PI3K signaling in the maintenance and viability of prostate cancer stem-like cell populations. Proc. Natl. Acad. Sci. USA.

[29] Tang H., Jin Y., Jin S., Tan Z., Peng Z., Kuang Y. (2016). Arsenite inhibits the function of CD133+ CD13+ liver cancer stem cells by reducing PML and Oct4 protein expression. Tumor Biol..

[30] Liang J., Slingerland J.M. (2003). Multiple roles of the PI3K/PKB (Akt) pathway in cell cycle progression. Cell Cycle.

[31] Osaki M., Oshimura M., Ito H. (2004). PI3K-Akt pathway: Its functions and alterations in human cancer. Apoptosis.

[32] Brunet A., Bonni A., Zigmond M.J., Lin M.Z., Juo P., Hu L.S., Anderson M.J., Arden K.C., Blenis J., Greenberg M.E. (1999). Akt promotes cell survival by phosphorylating and inhibiting a forkhead transcription factor. Cell.

[33] Tzivion G., Dobson M., Ramakrishnan G. (2011). FoxO transcription factors; Regulation by AKT and 14-3-3 proteins. Biochim. Biophys. Acta Mol. Cell Res..

[34] Katayama K., Nakamura A., Sugimoto Y., Tsuruo T., Fujita N. (2008). FOXO transcription factor-dependent p15INK4b and p19 INK4d expression. Oncogene.

[35] Gomis R.R., Alarcon C., He W., Wang Q., Seoane J., Lash A., Massague J. (2006). A FoxO-Smad synexpression group in human keratinocytes. Proc. Natl. Acad. Sci. USA.

[36] Seoane J., Le H., Shen L., Anderson S.A., Massague J. (2004). Integration of Smad and Forkhead Pathways in the Control of Neuroepithelial and Glioblastoma Cell Proliferation Joan. Cell.

[37] Medema R.H., Kops G.J.P.L., Bos J.L., Burgering B.M.T. (2000). AFX-like Forkhead transcription factors mediate cell-cycle regulation by Ras and PKB through p27(kip1). Nature.

[38] Knudsen E.S., Knudsen K.E. (2008). Tailoring to RB: Tumour suppressor status and therapeutic response. Nat. Rev. Cancer.

[39] Jiao W., Datta J., Lin H.M., Dundr M., Rane S.G. (2006). Nucleocytoplasmic shuttling of the retinoblastoma tumor suppressor protein via Cdk phosphorylation-dependent nuclear export. J. Biol. Chem..

[40] Rotili D., Mai A. (2011). Targeting histone demethylases: A new avenue for the fight against cancer. Genes Cancer.

[41] Ma S. (2016). Deciphering ZIC2/OCT4 signaling as a vulnerability in liver cancer stem cells. Transl. Cancer Res..

[42] Visvader J.E., Lindeman G.J. (2008). Cancer stem cells in solid tumours: Accumulating evidence and unresolved questions. Nat. Rev. Cancer.

[43] Hirschhaeuser F., Menne H., Dittfeld C., West J., Mueller-Klieser W., Kunz-Schughart L.A. (2010). Multicellular tumor spheroids: An underestimated tool is catching up again. J. Biotechnol..

[44] Pastrana E., Silva-Vargas V., Doetsch F. (2011). Eyes wide open: A critical review of sphere-formation as an assay for stem cells. Cell Stem Cell.

[45] Yamashita T., Wang X. (2013). Cancer stem cells in the development of liver cancer. J. Clin. Investig..

[46] Haraguchi N., Ishii H., Mimori K., Tanaka F., Ohkuma M., Kim H.M., Akita H., Takiuchi D., Hatano H., Nagano H. (2010). CD13 is a therapeutic target in human liver cancer stem cells. J. Clin. Investig..

[47] Lee T.K.W., Castilho A., Cheung V.C.H., Tang K.H., Ma S., Ng I.O.L. (2011). CD24 + Liver Tumor-Initiating Cells Drive Self-Renewal and Tumor Initiation through STAT3-Mediated NANOG Regulation. Cell Stem Cell.

[48] Lei Z.J., Wang J., Xiao H.L., Guo Y., Wang T., Li Q., Liu L., Luo X., Fan L.L., Lin L. (2015). Lysine-specific demethylase 1 promotes the stemness and chemoresistance of Lgr5+liver cancer initiating cells by suppressing negative regulators of β-catenin signaling. Oncogene.

[49] Ma S., Chan K.W., Hu L., Lee T.K.W., Wo J.Y.H., Ng I.O.L., Zheng B.J., Guan X.Y. (2007). Identification and Characterization of Tumorigenic Liver Cancer Stem/Progenitor Cells. Gastroenterology.

[50] Mima K., Okabe H., Ishimoto T., Hayashi H., Nakagawa S., Kuroki H., Watanabe M., Beppu T., Tamada M., Nagano O. (2012). CD44s regulates the TGF-β-mediated mesenchymal phenotype and is associated with poor prognosis in patients with hepatocellular carcinoma. Cancer Res..

[51] Yamashita T., Ji J., Budhu A., Forgues M., Yang W., Wang H.Y., Jia H., Ye Q., Qin L.X., Wauthier E. (2009). EpCAM-Positive Hepatocellular Carcinoma Cells Are Tumor-Initiating Cells with Stem/Progenitor Cell Features. Gastroenterology.

[52] Chang F., Lee J.T., Navolanic P.M., Steelman L.S., Shelton J.G., Blalock W.L., Franklin R.A., McCubrey J.A. (2003). Involvement of PI3K/Akt pathway in cell cycle progression, apoptosis, and neoplastic transformation: A target for cancer chemotherapy. Leukemia.

[53] Whittaker S., Marais R., Zhu A.X. (2010). The role of signaling pathways in the development and treatment of hepatocellular carcinoma. Oncogene.

[54] Martelli A.M., Evangelisti C., Follo M.Y., Ramazzotti G., Fini M., Giardino R., Manzoli L., McCubrey J.A., Cocco L. (2011). Targeting the Phosphatidylinositol 3-Kinase/Akt/Mammalian Target of Rapamycin Signaling Network in Cancer Stem Cells. Curr. Med. Chem..

[55] Wu Y., Zhang J., Zhang X., Zhou H., Liu G., Li Q. (2020). Cancer Stem Cells: A Potential Breakthrough in HCC-Targeted Therapy. Front. Pharmacol..

[56] Carmona F.J., Montemurro F., Kannan S., Rossi V., Verma C., Baselga J., Scaltriti M. (2016). AKT signaling in ERBB2-amplified breast cancer. Pharmacol. Ther..

[57] Nitulescu G.M., Van De Venter M., Nitulescu G., Ungurianu A., Juzenas P., Peng Q., Olaru O.T., Grǎdinaru D., Tsatsakis A., Tsoukalas D. (2018). The Akt pathway in oncology therapy and beyond (Review). Int. J. Oncol..

[58] Bellacosa A., Kumar C.C., Di Cristofano A., Testa J.R. (2005). Activation of AKT kinases in cancer: Implications for therapeutic targeting. Adv. Cancer Res..

[59] Fresno Vara J.Á., Casado E., de Castro J., Cejas P., Belda-Iniesta C., González-Barón M. (2004). P13K/Akt signalling pathway and cancer. Cancer Treat. Rev..

[60] Revathidevi S., Munirajan A.K. (2019). Akt in cancer: Mediator and more. Semin. Cancer Biol..

[61] Gener P., Rafael D., Seras-franzoso J., Perez A., Pindado L.A., Casas G., Arango D., Fernández Y., Díaz-riascos Z.V., Abasolo I. (2019). Pivotal role of AKT2 during dynamic phenotypic change of breast cancer stem cells. Cancers.

[62] Gargini R., Cerliani J.P., Escoll M., Antõn I.M., Wandosell F. (2015). Cancer stem cell-like phenotype and survival are coordinately regulated by Akt/FoxO/bim pathway. Stem Cells.

[63] Lee J., Kim M.S., Park S.H., Jang Y.K. (2018). Tousled-like kinase 1 is a negative regulator of core transcription factors in murine embryonic stem cells. Sci. Rep..

[64] Park S.H., Yu S.E., Chai Y.G., Jang Y.K. (2014). CDK2-dependent phosphorylation of Suv39H1 is involved in control of heterochromatin replication during cell cycle progression. Nucleic Acids Res..

[65] Lee J., Kim M.S., Kim M.A., Jang Y.K. (2016). Calmidazolium chloride inhibits growth of murine embryonal carcinoma cells, a model of cancer stem-like cells. Toxicol. Vitr..

[66] Cheng G.Z., Chan J., Wang Q., Zhang W., Sun C.D., Wang L.H. (2007). Twist transcriptionally up-regulates AKT2 in breast cancer cells leading to increased migration, invasion, and resistance to paclitaxel. Cancer Res..

[67] Bahmad H.F., Cheaito K., Chalhoub R.M., Hadadeh O., Monzer A., Ballout F., El-Hajj A., Mukherji D., Liu Y.N., Daoud G. (2018). Sphere-Formation Assay: Three-dimensional in vitro culturing of prostate cancer stem/Progenitor sphere-forming cells. Front. Oncol..

[68] Morrison B.J., Steel J.C., Morris J.C. (2012). Sphere Culture of Murine Lung Cancer Cell Lines Are Enriched with Cancer Initiating Cells. PLoS ONE.

