# Supplementary material for: JIB-04, a Pan-Inhibitor of Histone Demethylases, Targets Histone-Lysine-Demethylase-Dependent AKT Pathway, Leading to Cell Cycle Arrest and Inhibition of Cancer Stem-Like Cell Properties in Hepatocellular Carcinoma Cells
Source: Int J Mol Sci. 2022 Jul 11;23(14):7657. doi: 10.3390/ijms23147657 (PMC9322929; doi:10.3390/ijms23147657)
Supplement: Supplementary file 1 [file ijms-23-07657-s001.zip › ijms-1727001-supplementary.pdf]

International Journal of *Molecular Sciences*

MDPI

Article

JIB-04, a Pan-Inhibitor of Histone Demethylases, Targets Histone-Lysine-Demethylase-Dependent AKT Pathway, Leading to Cell Cycle Arrest and Inhibition of Cancer Stem-Like Cell Properties in Hepatocellular Carcinoma Cells

Jina Lee[1,2,†], Ji-Soo Kim[1,2,†], Hye-In Cho[1,2], So-Ra Jo[1,2] and Yeun-Kyu Jang[1,2,*]

[1] Department of Systems Biology, College of Life Science and Biotechnology, Yonsei University, Seoul 120-749, Korea.
[2] BK21 Yonsei Education & Research Center for Biosystems, Yonsei University, Seoul 120-749, Korea.
* Correspondence: ykjang@yonsei.ac.kr; Tel.: +82-2-2123-5654; Fax: +82-2-312-5657
† These authors contributed equally to this work.

Citation: Lee, J.; Kim, J.-S.; Cho, H.-I.; Jo, S.-R.; Jang, Y.-K. JIB-04, a Pan-Inhibitor of Histone Demethylases, Targets Histone-Lysine-Demethylase-Dependent AKT Pathway, Leading to Cell Cycle Arrest and Inhibition of Cancer Stem-like Cell Properties in Hepatocellular Carcinoma Cells. *Int. J. Mol. Sci.* **2022**, *23*, 7657. https://doi.org/10.3390/ijms23147657

Academic Editor: Isabel Fabregat

Received: 29 April 2022
Accepted: 8 July 2022
Published: 11 July 2022

Publisher's Note: MDPI stays neutral with regard to jurisdictional claims in published maps and institutional affiliations.

Copyright: © 2022 by the authors. Licensee MDPI, Basel, Switzerland. This article is an open access article distributed under the terms and conditions of the Creative Commons Attribution (CC BY) license (https://creativecommons.org/licenses/by/4.0/).

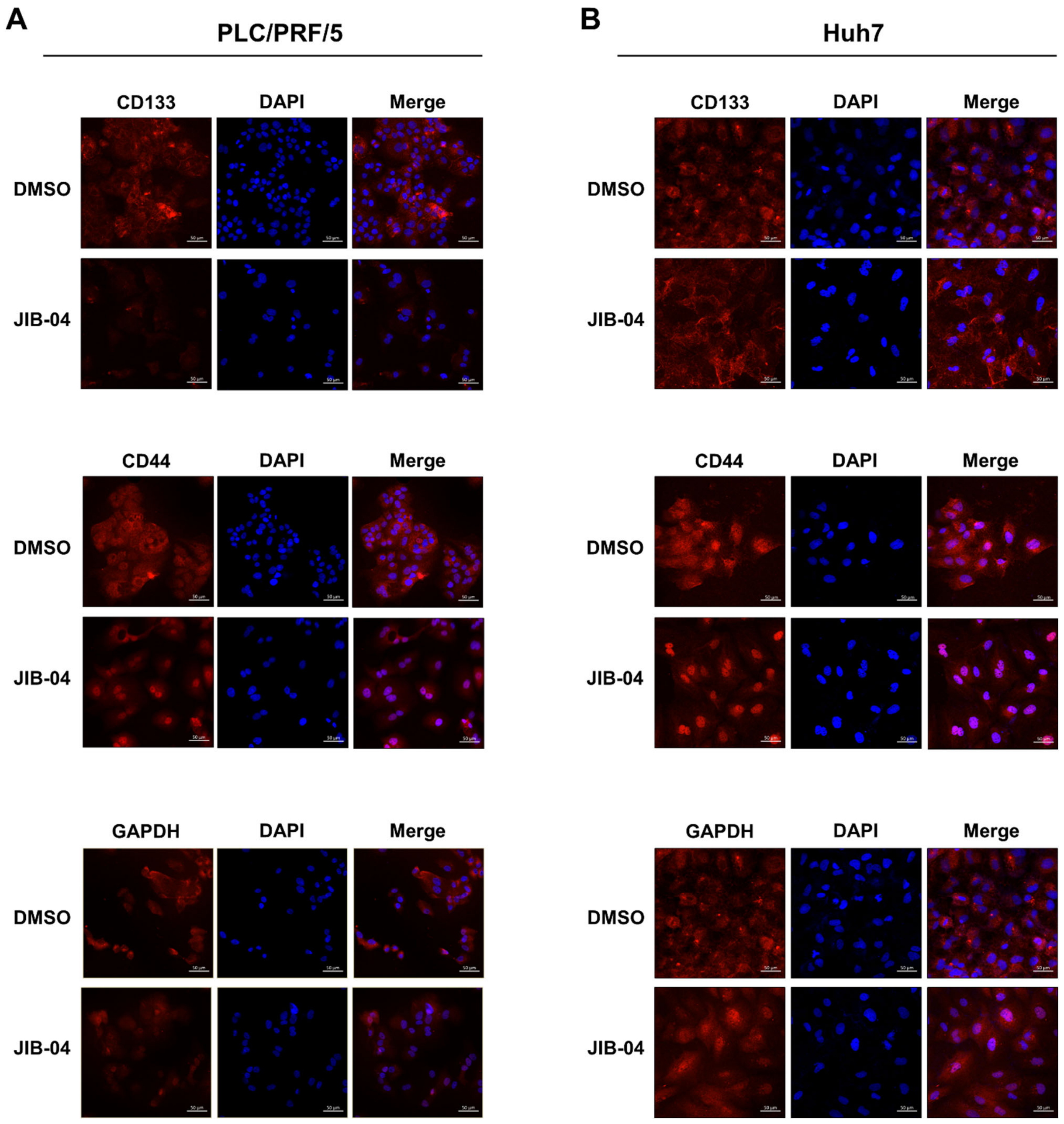

Supplementary Figure S1. The expression of cancer stem cell markers was decreased in JIB-04-treated HCC Cells. The fluorescent immunocytochemistry experiment showed that the expression of CD133 and CD44 was decreased in PLC/PRF/5 cells (A) and Huh7 cells (B). Representative images from two independent experiments are shown. Scale bar: 50 μM.

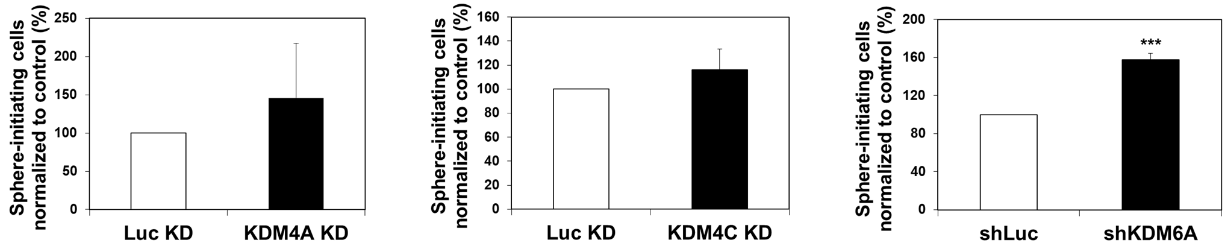

Supplementary Figure S2. KDM4A, KDM4C, and KDM6A are not required for the tumorsphere formation ability of CSCs in PLC/PRF/5 cells. CCK assay revealed that the viability of tumorsphere cells was increased in *KDM4A*-, *KDM4C*-, and *KDM6A*-depleted knockdown cells (n=3). Percentage of sphere-initiating cells measured by CCK assay. The number of control-knockdown cells (Luc KD) was set as 100. Data are represented as mean ± SEM of triplicate measurements. ***p <0.001.

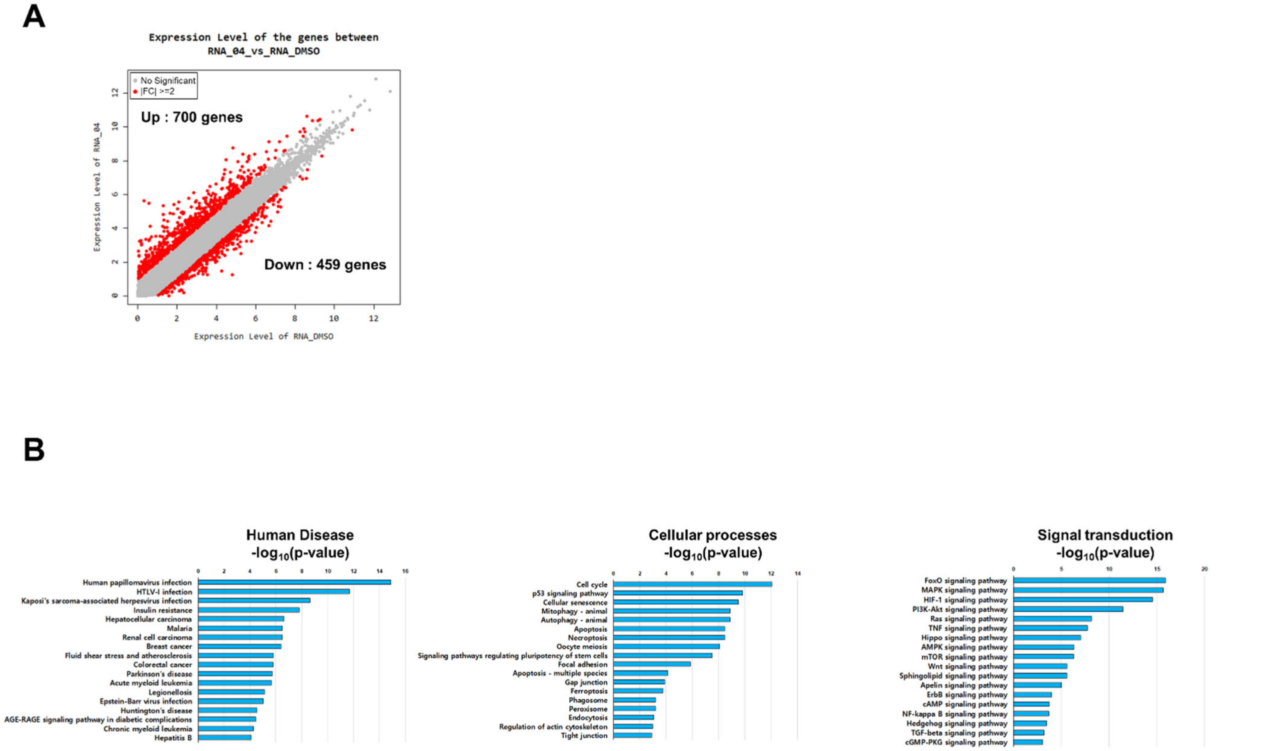

Supplementary Figure S3. Transcriptome analysis of PLC/PRF/5 cells after treatment with JIB-04. (A) Scatter plot of differential gene expression between cells treated with 6 μM JIB-04 or DMSO. (B) KEGG pathway enrichment analysis. Raw *p*-values were calculated by modified Fisher's exact test. The pathway enrichment scores (−Log10 *P*-value) among the differentially expressed genes are shown.

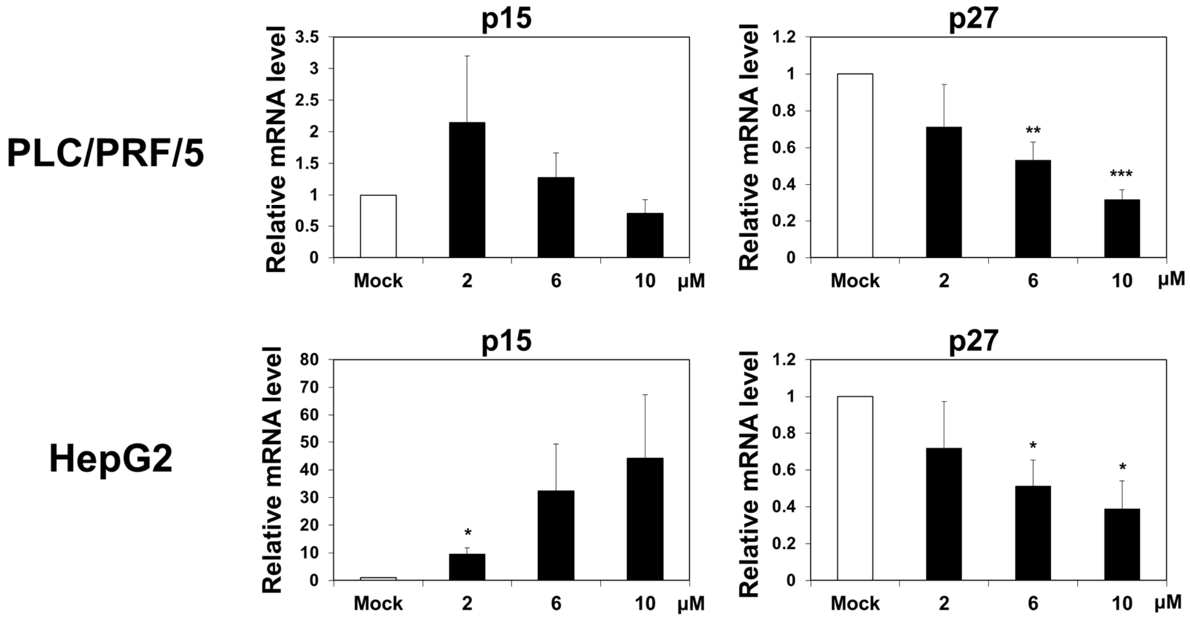

Supplementary Figure S4. The effect of JIB-04 treatment on the mRNA expression of CDK inhibitors such as *p15(Ink4b)* and *p27(kip1)*. The mRNA expression levels of *p15(Ink4b)* and *p27(kip1)* in PLC/PRF/5 and HepG2 cells cultured after treatment with increasing doses of JIB-04 or DMSO (mock control) were measured by qRT-PCR. All data are normalized to GAPDH and plotted relative to the expression level in control cells (n=3). Data are represented as mean ± SEM of triplicate measurements. *p <0.05, **p <0.01, and ***p <0.001.
